# Use of controlled nail dynamization technique for femoral shaft hypertrophic nonunion

**DOI:** 10.3389/fsurg.2025.1547793

**Published:** 2025-04-07

**Authors:** Qian Wu, Qi Wang, XueCheng Sun, Jun Liu, Gang Zhao, Ping Yu

**Affiliations:** Department of Orthopedics and Trauma Weifang People's Hospital, Shandong Second Medical University, Weifang, Shandong, China

**Keywords:** intramedullary nailing (IMN), femoral nonunion, nail dynamization, hypertrophic nonunion, poller screws

## Abstract

**Background:**

Femoral nonunion after intramedullary nailing (IMN) of a diaphyseal long bone fracture is a severe complication that requires effective management. The IMN dynamization has been used to treat hypertrophic nonunions previously. However, routine nail dynamization has only a low success rate and the risk of limb shortening.

**Methods:**

Two patients with femoral shaft fracture hypertrophic nonunion at 4 or 5 months after intramedullary nailing were treated with the therapeutic paradigm named “controlled nail dynamization”. In this paradigm, the interlocking nails are removed but the dynamic hole nails are retained. At the same time, four Poller screws were used to limit the movement of the intramedullary nail in the coronal and sagittal planes. The intramedullary nail can only generate compressive stress along the axial direction of the femoral shaft, thereby promoting fracture healing. So this technique was named “controlled nail dynamization”.

**Results:**

Here, we describe two cases of delayed healing of the femoral diaphysis, which were successfully treated through controlled nail dynamization. Followed up for more than 12 months. Bone union was achieved in both patients, and there were no complications such as nonunion and internal fixation failure.

**Conclusion:**

The controlled nail dynamization is feasible for safe and effective treatment for femoral shaft hypertrophic nonunion.

## Introduction

Intramedullary nailing (IMN) is considered the first-line treatment for femoral shaft fractures (1). The incidence of bone nonunion after intramedullary nailing is 6.3–12.5% (2). Dynamic intramedullary nailing, intramedullary nail replacement and adjuvant plates have been proposed as possible solutions to treat bone nonunion (especially for hypertrophic nonunion) and thus achieve mechanical stability of the fracture and improve structural stiffness.

Although there are multiple approaches to treat femoral shaft bone union after IMN, IMN dynamization is a quick, cost-effective, and effective way to promote healing (3). IMN dynamization is defined as removal of interlocking screws proximal or distal to the fracture site to allow bone compression at the fracture site. Previous studies on the efficacy of IMN dynamization to promote fracture healing reported mixed results, with some authors finding that dynamization was effective, while others reported low success rates and unacceptable fracture shortening. Evidence suggests that successful cure rates after dynamization of IMNs range from 19% to 82%, which may result from the lack of stability of IMNs during dynamization.

The basic principle of dynamization is to increase micromotion at the fracture gap, thereby stimulating fracture healing (4, 5). In one-third of unstable proximal or distal femoral shaft fractures, traditional dynamization of removed screws provides the required compression effect but may also lead to further sliding, rotation or angulation of the proximal or distal fracture fragments, generating an unsatisfactory dynamization effect. Thus, restricting the disordered movement of the IMN dynamization is the key to fracture healing.

The aim of this study is to introduce a therapeutic paradigm that increases IMN stability during IMN dynamism. For the first time, we used Poller screws to limit IMN sagittal and coronal movement in IMN dynamization and observed the clinical outcome of fracture healing.

## Surgical technique

**Figure 1:**
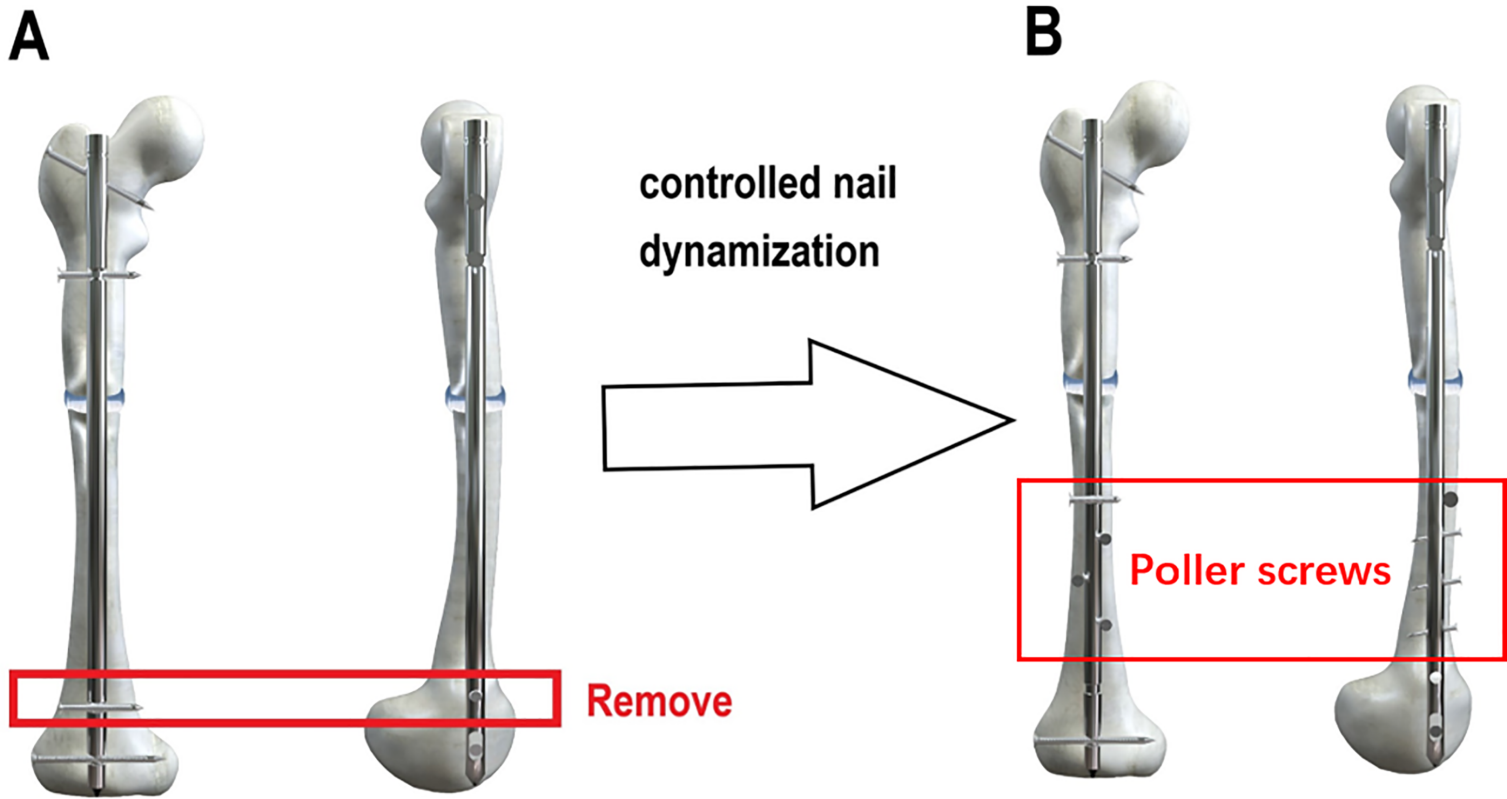
Schematic diagram of controlled nail dynamization techniques. **(A)** Static screws are removed, while dynamic screws are retained or added. **(B)** Poller screws are implanted in the medial, lateral, and/or posterior aspects of the intramedullary nail.

The medial, lateral and/or posterior parts of intramedullary nails were implanted with Poller screws. The stiffness of the medullary cavity could be increased by reducing its width. Meanwhile, the screws in the dynamic holes were retained or increased to restrict motion in the proximal or distal sagittal and coronal positions (Figure 1).

**Figure 2:**
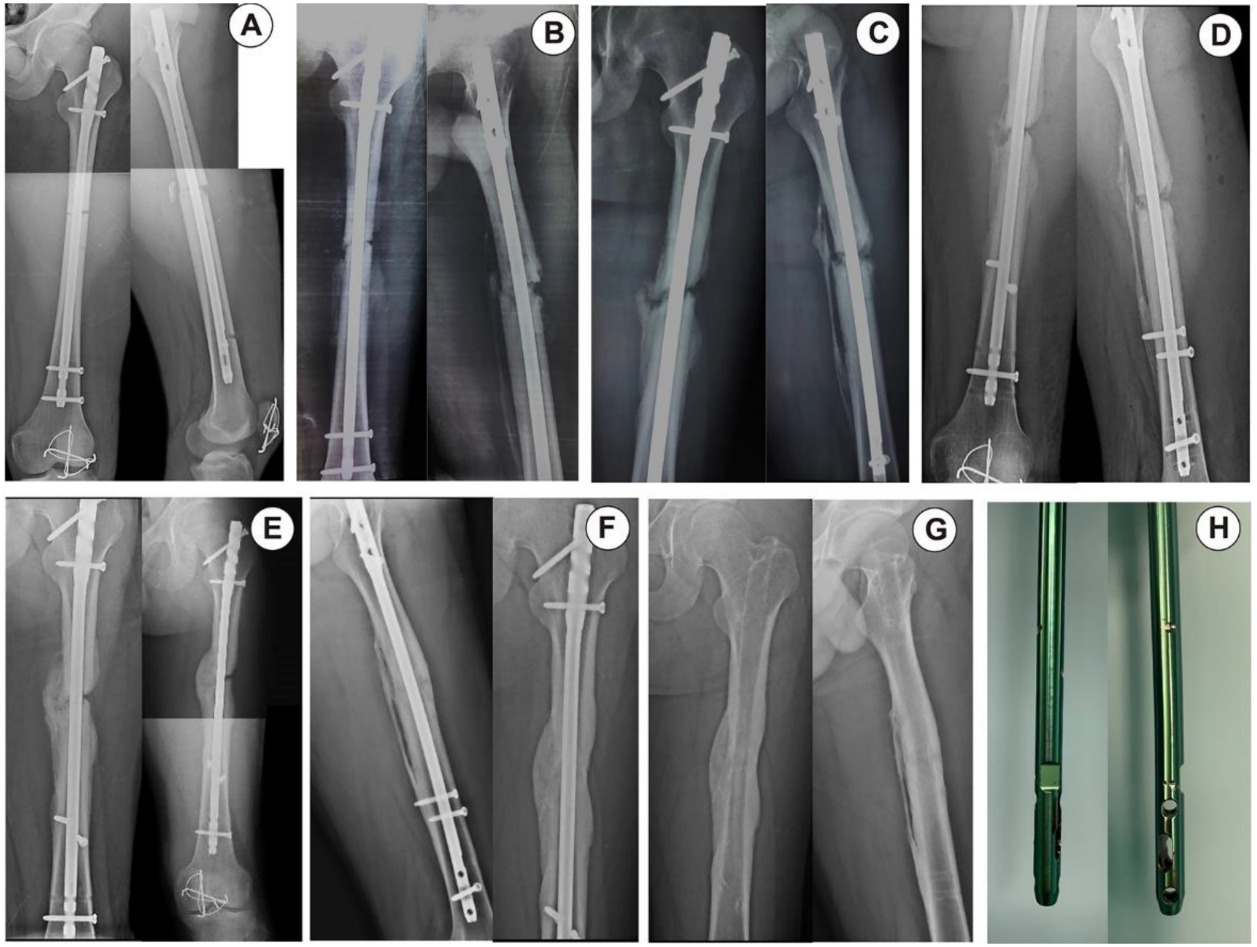
Case 1. **(A)** Immediately after the operation. **(B)** 6 weeks after the operation. **(C)** 18 weeks after the operation. **(D)** Controlled nail dynamization at 19 weeks after the operation. **(E)** 4 weeks after dynamization. **(F)** Full healing of the fracture at 24 weeks after dynamization. **(G,H)** Removal of intramedullary nails at 18 months after the operation.

**Figure 3:**
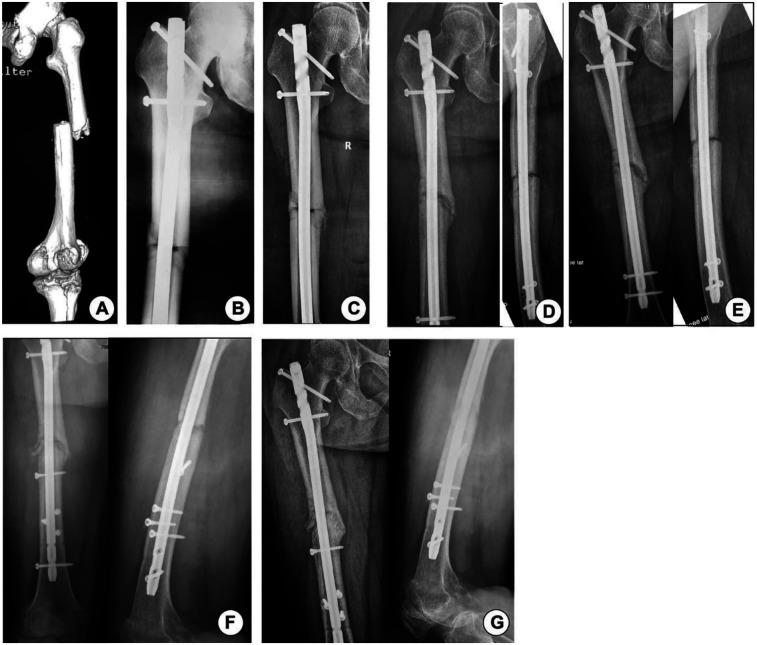
Case 2. **(A)** The position of the femur before the operation. **(B)** Immediately after the operation. **(C)** 4 weeks after the operation. **(D)** 8 weeks after the operation. **(E)** 16 weeks after the operation. **(F)** Immediately after controlled nail dynamization. **(G)** 8 weeks after dynamization.

## Case illustration

### Case 1

A 17-year-old male patient suffered a femoral shaft fracture due to traffic accident on a motorcycle, resulting in fracture of his femur and patella. The patient was a heavy smoker and suffered a 32-B2-type fracture (AO Foundation/Orthopaedic Trauma Association (AO/OTA) classification). Closed reduction intramedullary nailing was adopted in the operation. Reexamination at 2 months, 4 months and 6 months after surgery revealed unsatisfactory and significant delayed union of the fracture. Hypertrophic callus hyperplasia was found at the fractured end of the bone. Due to the instability of the fracture end, dynamization treatment was applied by removing the original static locking nails and placing dynamic locking nails, as well as Poller screw placement to prevent coronal displacement. In the rehabilitation process, the patient was instructed to carry out weight-bearing activities using the walking aid to stimulate healing of the fracture end. At a postoperative follow-up, significant callus healing had occurred at 2 months after the operation, and the fracture line disappeared after 4 months (Figure 2).

### Case 2

A 58-year-old female patient presented with a fracture of the right femoral shaft with a 32-B2-type fracture resulting from a bicycle accident. The patient underwent surgery with open reduction and intramedullary nailing at a Class 1 hospital. The patient underwent an outpatient follow-up intermittently after the operation, and fracture healing was unsatisfactory 5 months later. The patient visited our hospital for treatment. x-rays showed callus hyperplasia and a clear fracture line, as confirmed by computed tomography (CT), indicating that the fracture end was unstable. The patient underwent surgery, and the static nails were removed. Three anteroposterior Poller screws were placed at the distal end of the fracture, and a coronal nail was placed laterally to ensure the stability of intramedullary nailing. After the operation, the patient was urged to carry out weight-bearing activities to promote fracture healing. At a subsequent follow-up visit, x-rays showed significant changes in union of the fracture, with bony union achieved 2 months later (Figure 3).

## Discussion

Dynamic intramedullary nailing is an effective method to treat delayed union of the femur. However, some scholars have suggested that dynamic intramedullary nailing may aggravate the instability of the fracture end, which is not conducive to fracture healing. Therefore, understanding the mechanisms by which dynamic intramedullary nailing affects fracture healing, intuitively determining the optimal timing for dynamic intramedullary nailing and using a reasonable dynamic intramedullary nailing method help improve the fracture healing rate and reduce complications. Dynamic intramedullary nailing can increase micromotion and loading at the site of fractures with delayed union and improve the biomechanical environment of the fracture site. A study shows that reduced fracture gaps, enhanced vascularization of the fracture area, increased bone callus production, increased bone callus hardness and other changes are observed after the dynamic intramedullary nailing, with improved fracture healing. The promotion of fracture healing by dynamic intramedullary nailing may be related to the expression of multiple cytokines, directed differentiation of mesenchymal stem cells (MSC) and altered levels of cellular oxygen uptake in the fracture area (6, 7).

In the past, nail dynamization was routinely performed on all statically locked nails 2–4 months after surgery. Further evidence revealed that this dynamization was unnecessary for fracture healing and was subsequently unpopular as standard practice (8). There are several reasons for this shift in perception. First, evidence indicates that conventional motility is unnecessary for fracture healing and is associated with a risk of shortening, especially in spiral and long oblique fractures. In 1997, Wu (9) studied the efficacy of dynamic therapy for delayed union and nonunion of femur in 24 cases. They found a similar success rate to the current study at 58 percent, and also noted a greater than 20 percent rate of fractures shortening by more than 2 cm. Several studies have suggested the risk of shortening due to dynamization. Spiral, long oblique, and comminuted fractures are at greatest risk of shortening. Furthermore, in rotationally unstable fracture patterns, there is a risk of rotational malunion following removal of interlocking screws (10, 11).

Two dynamization methods are currently used, namely, “remove all locking screws at one end” (full dynamization) and “retain screws in the dynamic locking hole” (partial dynamization). Interlocking screws help maintain length and rotational stability. Removal of interlocking screws promotes fracture collapse and subsequent healing, and also allows rotation of the fracture site. In the former method, the locking screws on one side are completely removed, generating axial compression movement between fracture ends, which creates favorable conditions for fracture healing. However, the fracture end has also lost its stability and is prone to over compression, causing shortening and uncontrolled oscillation. In the latter method, the retained nails are confined to the dynamic locking hole. A certain degree of axial compression movement without rotation and bending is evident at the fracture end. However, the distal end of the intramedullary nail is still unstable in the coronal position. Therefore, we propose unrestricted swinging of the intramedullary nail at the coronal position with Poller screws; we call this technique controlled dynamization and have achieved satisfactory results. Based on the above considerations, we believe that the failure of intramedullary nail dynamization may be due to excessive dynamization, that is, the dynamics are not controlled dynamization that is effective for fracture healing. In the controlled dynamic technique, we limit the IMN to only produce axial compressive stress on the fracture end of the femoral shaft that is beneficial to fracture healing through the Poller screw. Through the treatment of two cases of femoral shaft hypertrophy nonunion, it was found that it can significantly promote fracture healing.

Femoral nonunion includes hypertrophic and atrophic types. The authors believe that controlled dynamization is only applicable to hypertrophic nonunions. Our opinion is supported by previous research. Previous studies have shown that callus diameter and open fracture have been identified as factors associated with IMN dynamization success and failure, respectively. Callus diameter is also used to measure the biological environment of the fracture site (12). These findings underscore the importance of blood supply for fracture healing. Open fractures deactivate most of the bone and surrounding soft tissue critical for fracture healing, predisposing to atrophic nonunions. Notably, in the current study, the lack of callus formation was predictive of nail dynamization failure (13).

Furthermore, the timing of dynamization is an important factor affecting therapeutic efficacy (14). Both early and late dynamization can have a detrimental effect on fracture healing. However, no uniform standard has been established for the optimal timing to perform dynamization of long bone intramedullary nails. For fractures of the tibia and femur or osteochondrosis, dynamization will produce a better result and thus allow dynamic intramedullary nailing at 10–24 weeks after initial intramedullary nailing. Additionally, the canal-diaphysis ratio (CDR) can help determine the timing of dynamization (14, 15).

In summary, the controlled nail dynamization technique, which incorporates Poller screws to restrict coronal and sagittal plane motion while preserving axial compression, demonstrates a promising approach for managing femoral shaft hypertrophic nonunion. However, the small sample size and limited follow-up duration necessitate further validation through larger multicenter studies and long-term evaluations. In recent years, design innovations in elastic self-locking intramedullary nails have provided novel insights into enhancing the stability of fracture fixation. Putame et al. (16) conducted numerical analyses to compare the mechanical performance of three elastic self-locking intramedullary nails.Their findings revealed that the Terzini-Putame nail exhibited a significantly higher stability index compared to conventional designs. Future research should explore integrating the design principles of elastic self-locking nails (e.g., enhanced axial stability) with controlled dynamization techniques. Such integration could further reduce reliance on auxiliary screws while minimizing the risk of complications associated with excessive dynamization.

## Data Availability

The original contributions presented in the study are included in the article/Supplementary Material, further inquiries can be directed to the corresponding author.
